# The Nucleoid Occlusion Protein SlmA Binds to Lipid Membranes

**DOI:** 10.1128/mBio.02094-20

**Published:** 2020-09-01

**Authors:** Miguel Ángel Robles-Ramos, William Margolin, Marta Sobrinos-Sanguino, Carlos Alfonso, Germán Rivas, Begoña Monterroso, Silvia Zorrilla

**Affiliations:** a Centro de Investigaciones Biológicas Margarita Salas, Consejo Superior de Investigaciones Científicas (CSIC), Madrid, Spain; b Department of Microbiology and Molecular Genetics, McGovern Medical School, University of Texas, Houston, Texas, USA

**Keywords:** *E. coli*, FtsZ, SlmA, fluorescence microscopy, lipid membranes, membrane binding, microfluidics, nucleoid occlusion

## Abstract

Protection of the chromosome from scission by the division machinery during cytokinesis is critical for bacterial survival and fitness. This is achieved by nucleoid occlusion, which, in conjunction with other mechanisms, ensures formation of the division ring at midcell. In Escherichia coli, this mechanism is mediated by SlmA, a specific DNA binding protein that antagonizes assembly of the central division protein FtsZ into a productive ring in the vicinity of the chromosome. Here, we provide evidence supporting direct interaction of SlmA with lipid membranes, tuned by its binding partners FtsZ and SlmA binding sites (SBS) on chromosomal DNA. Reconstructions in minimal membrane systems that mimic cellular environments show that SlmA binds to lipid-coated microbeads or locates at the edge of microfluidic-generated microdroplets, inside which the protein is encapsulated. DNA fragments containing SBS sequences do not seem to be recruited to the membrane by SlmA but instead compete with SlmA’s ability to bind lipids. The interaction of SlmA with FtsZ modulates this behavior, ultimately triggering membrane localization of the SBS sequences alongside the two proteins. The ability of SlmA to bind lipids uncovered in this work extends the interaction network of this multivalent regulator beyond its well-known protein and nucleic acid recognition, which may have implications in the overall spatiotemporal control of division ring assembly.

## Abstract (precis)

Successful bacterial proliferation relies on the spatial and temporal precision of cytokinesis and its regulation by systems that protect the integrity of the nucleoid. In Escherichia coli, one of these protectors is SlmA protein, which binds to specific DNA sites around the nucleoid and helps to shield the nucleoid from inappropriate bisection by the cell division septum. Here, we discovered that SlmA not only interacts with the nucleoid and septum-associated cell division proteins but also binds directly to cytomimetic lipid membranes, adding a novel putative mechanism for regulating the local activity of these cell division proteins. We find that interaction between SlmA and lipid membranes is regulated by SlmA’s DNA binding sites and protein binding partners as well as chemical conditions, suggesting that the SlmA-membrane interactions are important for fine-tuning the regulation of nucleoid integrity during cytokinesis.

## INTRODUCTION

Reproduction of bacillary bacteria requires duplication of the chromosome, segregation of the two copies generated, and division of the whole cell into two halves. This cascade of events is tightly regulated in time and space through the concurrence of several mechanisms (1). One of these mechanisms is nucleoid occlusion, which, in Escherichia coli, depends on SlmA, a DNA binding protein that counteracts division ring formation nearby the chromosome to protect this key element against damages inflicted by the division machinery (2). To accomplish this task, SlmA disrupts the GTP-dependent polymerization of the central division protein FtsZ, the scaffold on which the division ring is built (3–5). Jointly with other systems, acting either as agonists or as antagonists of FtsZ assembly, SlmA participates in the events leading to FtsZ ring formation, specifically at the cell center, also aiding in the coordination of chromosome segregation and cytokinesis (6, 7).

After its identification, two independent groups found that SlmA is a sequence-specific DNA binding protein targeting a 20-bp sequence (SlmA binding sequence [SBS]), repeated over 20 times along the chromosome with the sole exception of the Ter region (4, 8). The absence of SBS sites in the Ter macrodomain, which is the last portion of the chromosome that is segregated away from midcell, contributes to the local depletion of SlmA, allowing normal FtsZ polymerization and initiation of FtsZ ring assembly during the last stages of chromosome replication and segregation (4). SlmA binds to the SBS sequences through a helix-turn-helix (HTH) motif located in its N-terminal domain, resulting in complexes with four SlmA monomers per SBS site, the affinity of which is strongly dependent on ionic strength (5, 8, 9). Aside from its interaction with DNA, SlmA self-associates into dimers mediated by hydrophobic contacts involving residues within its C-terminal region, maintained in the complex with the SBS such that the four monomers binding each site are actually a pair of dimers (8, 9). In addition, the nucleoprotein complexes of SlmA directly interact with FtsZ through two different regions of the latter, a conserved ∼20-amino-acid (aa) sequence at the C terminus and a second site within its globular domain (10). Both the polymers cooperatively formed by FtsZ in the presence of GTP and the shorter oligomers of the protein observed when bound to GDP are recognized by SlmA (5, 9). One of the consequences of these interactions is the promotion of FtsZ polymer disassembly (4, 5, 10) without altering the GTPase activity of FtsZ within its single-stranded protofilaments (5, 9). The antagonistic action of SlmA on FtsZ assembly is likely reinforced by the spreading of SlmA subunits on DNA adjacent to the SBS sites (9).

It is widely accepted that the blockage of FtsZ ring assembly around the nucleoid by SlmA is not limited to the cytoplasm but should also occur at noncentral regions of the membrane (6, 7, 9, 10). Such restrictions would reinforce the inhibition by other antagonists, like the Min system, which acts specifically at the membrane near the cell poles (11, 12). Along these lines, Noc, the protein effecting nucleoid occlusion in Bacillus subtilis, has been reported to bind to the membrane, and the formation of large nucleoprotein complexes at the membrane has been postulated to aid in the spatial regulation of divisome assembly (13). Lacking evidence of a direct interaction of SlmA with the membrane, different mechanisms have been proposed to explain how this protein could exert its effects on membrane-associated FtsZ. The prevalence of genes coding for membrane proteins close to the SBS sites targeted by SlmA suggested that transertion (coupled transcription, translation, and insertion) of nascent membrane proteins, encoded by these sequences, could be a possible mechanism to bring the SBS sites and SlmA concomitantly to the membrane (9). More recently, it has been observed that under conditions resembling the crowded nature of the cytoplasm, multivalent complexes consisting of FtsZ, SlmA, and DNA carrying a specific SBS sequence form dynamic biomolecular condensates in which FtsZ remains active for GTP-induced polymerization (14). Interestingly, these FtsZ-SlmA-SBS condensates preferentially locate at the membrane when reconstructed in biomimetic compartmentalized microdroplets stabilized by lipids (14).

Here, we report that SlmA binds directly to lipid membranes, employing an approach that involves different minimal membrane systems, and analyze the principal factors modifying this recognition by using biochemical, biophysical, and imaging methods. We also evaluate how the other two elements recognized by SlmA, FtsZ and the SBS on DNA, modulate these newly identified membrane interactions. We propose that the observed tendency of SlmA to bind to lipid membranes may cooperate with other previously suggested mechanisms, principally transertion and biomolecular condensation, to target SlmA to the membrane, with the aim of hindering FtsZ ring formation at noncentral areas of the cell.

## RESULTS

### SlmA binds to biomimetic lipid membranes.

**FIG 1:**
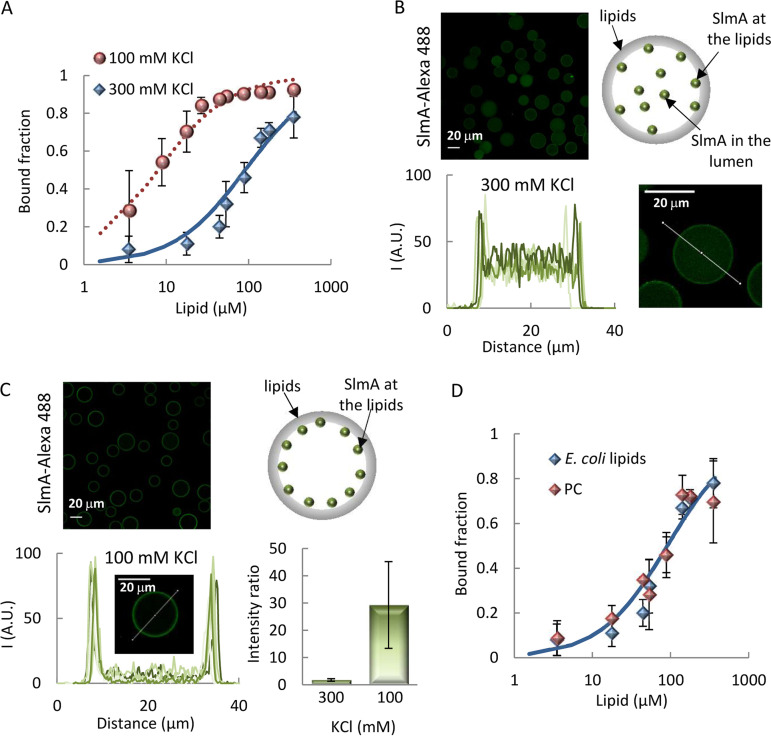
Interaction of SlmA with lipids. (A) Fraction of SlmA bound to microbeads coated with the E. coli lipid mixture as a function of the accessible lipid concentration in working buffer with different KCl concentrations. Solid line represents the fit of the model indicated in the main text to the experimental data, with *c*_50_ = 91 ± 15 μM. Dotted line corresponds to a simulation, using the same model and *c*_50_ = 8 μM. Encapsulation of SlmA inside microfluidics microdroplets stabilized by the E. coli lipid mixture in working buffer with 300 mM KCl (B) or 100 mM KCl (C), with representative confocal images of encapsulated SlmA, schematics illustrating the distribution of SlmA within the microdroplets (right), and intensity profiles of 5 different microdroplets corresponding to the green channel, obtained along the line as drawn in the images. The ratios of the intensity at the membrane to that at the lumen, corresponding to the average ± SD are also shown (*n* = 5, 300 mM KCl; *n* = 15, 100 mM KCl). The total concentration of SlmA was 5 μM, with a tracer concentration of 1 μM. (D) Fractions of SlmA bound to microbeads coated with the E. coli lipid mixture or with PC as a function of the accessible lipid concentration in working buffer with 300 mM KCl. Solid line as described for panel A. In panels A and D, the concentration of SlmA-Alexa 488 was 250 nM, and data are the averages from 3 (E. coli lipids) or 2 (PC) independent experiments ± SDs.

To determine if purified SlmA could directly bind lipids, we followed a strategy previously applied to other division proteins, making use of microbeads coated with a lipid mixture matching the composition of the E. coli inner membrane (15, 16). Titrations of SlmA labeled with Alexa Fluor 488 (SlmA-Alexa 488) with increasing concentration of the lipid-coated microbeads, in working buffer with 300 mM KCl, showed that a significant and gradually higher fraction of the protein pelleted alongside the microbeads (Fig. 1A). Analysis of the binding curve obtained using a Langmuir isotherm, with no assumption about the stoichiometry of the interaction, rendered binding of the 50% of total amount of protein at a lipid concentration (*c*_50_) of 91 ± 15 μM. Binding of SlmA to lipid membranes was independently confirmed by using biolayer interferometry (see Fig. S1A in the supplemental material). Addition of protein to tips coated with the E. coli lipid mixture showed a concentration-dependent rise in the signal, as expected for binding (Fig. S1B).

10.1128/mBio.02094-20.1 FIG S1 Binding of SlmA to lipids by biolayer interferometry. (A) Representative profiles of the binding of SlmA to E. coli lipids at the indicated protein concentrations. (B) Variations of the response at equilibrium with SlmA concentration in working buffer with 300 mM KCl. Data are the averages from at least 2 independent experiments ± SDs. Download FIG S1, TIF file, 0.8 MB. Copyright © 2020 Robles-Ramos et al. 2020 Robles-Ramos et al. This content is distributed under the terms of the Creative Commons Attribution 4.0 International license.

SlmA, with SlmA-Alexa 488 as a tracer, was subsequently encapsulated inside microdroplets generated by microfluidics stabilized by the E. coli lipid mixture (see Fig. S2). At 300 mM KCl, part of the protein was found at the edges of the microdroplets, with a significant fraction homogeneously distributed in the lumen, as observed in the intensity profiles obtained across the droplets (Fig. 1B). This result further evidences the tendency of the protein to interact with lipid surfaces.

10.1128/mBio.02094-20.2 FIG S2 Schematics of the microfluidic procedure for the formation of microdroplets and conversion of those microdroplets into giant unilamellar vesicles. The latter shows the step through which the microdroplets acquire the outer lipid layer. Encapsulated protein(s), with or without the SBS sequences, is in working buffer with KCl. Download FIG S2, TIF file, 1.4 MB. Copyright © 2020 Robles-Ramos et al. 2020 Robles-Ramos et al. This content is distributed under the terms of the Creative Commons Attribution 4.0 International license.

Next, the impact of ionic strength on SlmA binding to lipid membranes was studied. Increasing the concentration of KCl to 500 mM and even 1 M had a minor impact on protein binding in the experiments with lipid-coated microbeads with respect to that determined at 300 mM KCl (see Fig. S3A). Interestingly, decreasing the concentration of KCl to 100 mM resulted in a remarkable shift of the binding curve toward lipid concentrations around an order of magnitude lower (Fig. 1A). This large effect on SlmA binding was also detected in microfluidics encapsulation experiments. At this salt concentration, virtually all SlmA was localized at the lipid monolayer of the microdroplets, according to the intensity profiles retrieved (Fig. 1C). This protein localization pattern was analogous to that when encapsulated inside giant vesicles (see Fig. S4), obtained by off-chip conversion of microdroplets through the acquisition of a second lipid layer using an adapted droplet transfer method (Fig. S2). The presence of a crowder inside the microdroplets, required for the generation of giant unilamellar vesicles (GUVs), did not modify the protein distribution under these encapsulation conditions (Fig. S4).

10.1128/mBio.02094-20.3 FIG S3 Fraction of SlmA bound to microbeads coated with different lipids. Effect of KCl on binding to E. coli lipids (EcL) (A) or phosphatidylcholine (PC) (B) as a function of the accessible lipid concentration in working buffer. (C) Comparison between both lipids at two different concentrations. In all experiments, the concentration of SlmA-Alexa 488 was 250 nM and data are the average from 3 (EcL) or 2 (PC) independent experiments ± SDs. Download FIG S3, TIF file, 2.6 MB. Copyright © 2020 Robles-Ramos et al. 2020 Robles-Ramos et al. This content is distributed under the terms of the Creative Commons Attribution 4.0 International license.

10.1128/mBio.02094-20.4 FIG S4 Encapsulation of SlmA inside microfluidics microdroplets stabilized by the E. coli lipid mixture (A) and vesicles formed from them (B) in working buffer with 100 mM KCl and 150 g/liter Ficoll. Total SlmA concentration was 5 μM (with 1 μM tracer). Intensity profiles correspond to the signal of the green channel obtained along the line depicted in the images. Download FIG S4, TIF file, 1.9 MB. Copyright © 2020 Robles-Ramos et al. 2020 Robles-Ramos et al. This content is distributed under the terms of the Creative Commons Attribution 4.0 International license.

The ability of SlmA to bind to lipid membranes composed solely of neutral lipids was also tested through assays using microbeads coated with phosphatidylcholine (PC) (Fig. 1D). Significant interaction was also found in this case and, at 300 mM KCl, the binding curve was equivalent within error to that determined with the E. coli lipid mixture under the same conditions. As with those, binding to PC was relatively insensitive to salt in the 300 mM to 1 M range, whereas at 100 mM KCl, the interaction was strongly favored (Fig. S3B and C). This suggests that hydrophobic interactions play a role in the lipid recognition by SlmA.

From all these experiments, it can be concluded that SlmA binds to lipid membranes when reconstructed in cell mimetic systems. Evolution of the apparent affinity of the interaction with salt follows a mixed trend, being strongly enhanced at low concentrations but weakly affected above a certain threshold. Moreover, hydrophobic forces also seem to contribute to the overall interaction scheme.

### Specific SBS sequences compete with lipid membranes for binding to SlmA.

**FIG 2:**
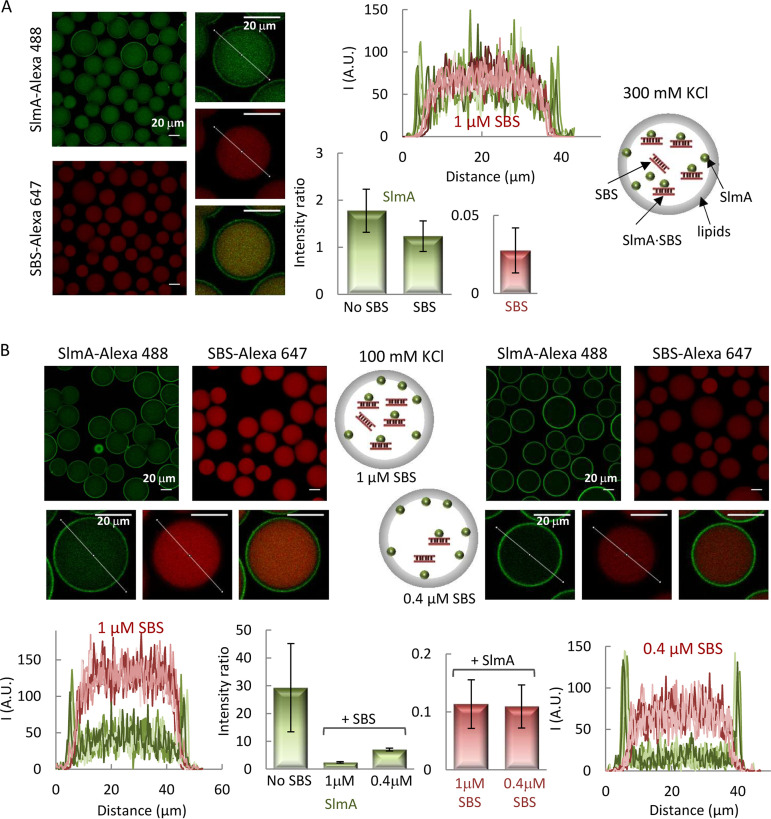
Encapsulation of SlmA and SBS in microfluidics microdroplets stabilized by the E. coli lipid mixture. Encapsulation in working buffer with 300 mM KCl (A) or 100 mM KCl (B). Shown are representative confocal microscopy images of the microdroplets in the green and red channels, including merged images of single microdroplets, and intensity profiles corresponding to 5 different microdroplets, obtained along the line as drawn in the images, in each channel. The ratios of the intensity at the membrane to that at the lumen, corresponding to the average ± SD, are also shown, together with those of SlmA encapsulated alone under the same buffer conditions for comparison. The concentration of SlmA was 5 μM, with 1 μM tracer. The concentrations of SBS tracer were 1 μM (A and B, left) or 0.4 μM (B, right). Schematics illustrating the distribution of SlmA and SBS within the microdroplets are shown.

Next, we evaluated the impact of the SBS sequences specifically recognized by SlmA on the interaction of the protein with the membrane. To this end, we coencapsulated SlmA, with SlmA-Alexa 488 as a tracer, and a double-stranded SBS sequence containing a single SlmA binding site, labeled with Alexa Fluor 647 (SBS-Alexa 647), inside microfluidics microdroplets stabilized by the E. coli lipid mixture. Under all conditions assayed, the signal arising from the red-labeled DNA evidenced its homogeneous distribution inside the microdroplets, with the intensity in the profiles dropping to basal levels at the lipid boundary (Fig. 2). SlmA, in turn, partitioned between the lumen and the membrane to a greater or lesser extent depending on the SBS and salt concentration. At 300 mM KCl, the signal corresponding to SlmA at the edges of the microdroplets was similar to that in the lumen (Fig. 2A), in contrast with the slightly higher relative signal observed at the membrane in the absence of the SBS (cf. Fig. 1B and 2A). This suggests competition between the SBS and the membrane for binding to SlmA.

Competition was more obvious in the experiments conducted at 100 mM KCl, in which two different SBS concentrations were tested. At 1 μM SBS, part of the protein dislodged from the lipid boundary (Fig. 2B, left) compared with the high levels of SlmA at this location in the absence of SBS (Fig. 1C), shifting toward the lumen of the microdroplets. Accordingly, the intensity in the green channel in that region of the droplet significantly increased, overlapping with the signal corresponding to the red-labeled SBS (Fig. 2B, left). SlmA detachment from the membrane was subtler at 0.4 μM SBS (Fig. 2B, right), which is compatible with a concentration-dependent competition between the two types of ligands for the protein. There was no evidence of recruitment of the SBS to the lipid membrane, even when the complex was already formed in solution before encapsulation, despite the significant fraction of SlmA remaining at the edge of the microdroplets, further supporting the competition between both ligands.

**FIG 3:**
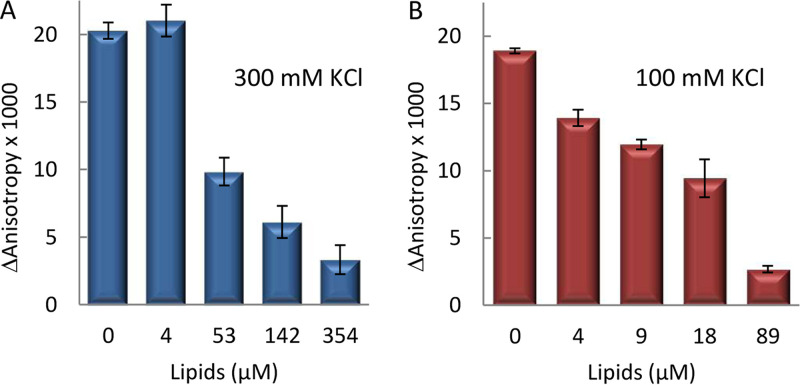
Competition between SBS and lipids for SlmA binding. Shown is the enhancement of fluorescence anisotropy in the presence of SlmA with respect to the value for the free SBS-Fl, as a function of the concentration of accessible lipids coating microbeads, in working buffer with the specified KCl concentrations. The concentrations of SlmA were 250 nM (A) and 125 nM (B), and that of SBS-Fl was 62.5 nM. Anisotropy was measured after microbead pelleting. Data are the averages from 3 independent experiments ± SDs.

We also studied the impact of SBS-containing DNA on lipid binding by SlmA through fluorescence anisotropy measurements on samples with fluorescein-labeled SBS (SBS-Fl), unlabeled SlmA, and increasing concentrations of E. coli lipid-coated microbeads after pelleting the microbeads by centrifugation (Fig. 3). The concentrations of SBS and SlmA in these experiments were kept constant. As expected, in the absence of lipids, the anisotropy of free SBS-containing DNA increased upon addition of SlmA (5) (Fig. 3). Inclusion of lipid-coated microbeads resulted in a reduction in the retrieved anisotropy that was dependent on lipid concentration, both at 100 and at 300 mM KCl (Fig. 3A and B). This is compatible with a release of the protein from the nucleoprotein complex upon lipid recognition, in good agreement with that observed in the microdroplet assays. The total intensity measured for the labeled SBS after microbead sedimentation in these experiments was largely independent of the concentration of microbeads, providing additional evidence of a lack of ternary complexes involving both DNA and lipids.

### FtsZ promotes the recruitment of SBS to the membrane by SlmA.

**FIG 4:**
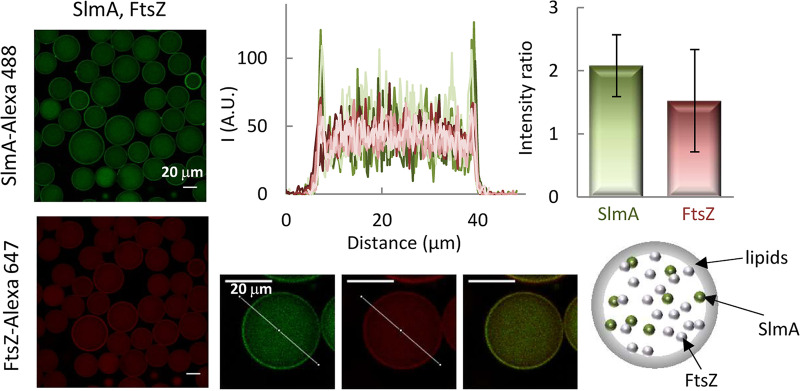
Encapsulation of SlmA and FtsZ inside microdroplets stabilized by the E. coli lipid mixture. Representative confocal images are shown with intensity profiles in the green and red channels corresponding to 5 different microdroplets, obtained along the lines depicted in the images, including the merged image of a single microdroplet. The ratios of the intensity at the membrane to that at the lumen, corresponding to the averages ± SDs, are also shown. A schematic illustrating the distribution of the two elements in the microdroplets is shown. The concentrations of SlmA and FtsZ were 5 and 12 μM, respectively, with 1 μM tracers, in working buffer with 100 mM KCl.

The direct recognition of FtsZ, favored by SBS binding, is central to the interaction network of SlmA. To determine the effect of FtsZ on the binding of SlmA to the membrane, we simultaneously encapsulated both proteins inside microdroplets stabilized by the E. coli lipid mixture in working buffer with 100 mM KCl (Fig. 4). The inclusion of FtsZ resulted in a significant shift of a fraction of SlmA from its typical location at the membrane (cf. Fig. 1C) to the lumen of the microdroplet, presumably due to the interaction between the two proteins. Indeed, the intensity signals of SlmA-Alexa 488 and of FtsZ labeled with Alexa Fluor 647 (FtsZ-Alexa 647) overlapped both inside the microdroplet and at the lipid boundary, reflecting an analogous distribution pattern (Fig. 4). Encapsulation of FtsZ alone showed that although most of the protein remained in the lumen, part of it was also found at the lipid interface (see Fig. S5). This is consistent with the previously described low tendency of FtsZ to bind to microbeads coated with E. coli lipids under these buffer conditions (17).

10.1128/mBio.02094-20.5 FIG S5 Encapsulation of FtsZ inside microfluidics microdroplets stabilized by the E. coli lipid mixture. Total FtsZ concentration was 12 μM (with 1 μM tracer) in working buffer with 100 mM KCl. Intensity profile corresponds to the signal of the red channel obtained along the line depicted in the image. Download FIG S5, TIF file, 0.5 MB. Copyright © 2020 Robles-Ramos et al. 2020 Robles-Ramos et al. This content is distributed under the terms of the Creative Commons Attribution 4.0 International license.

**FIG 5:**
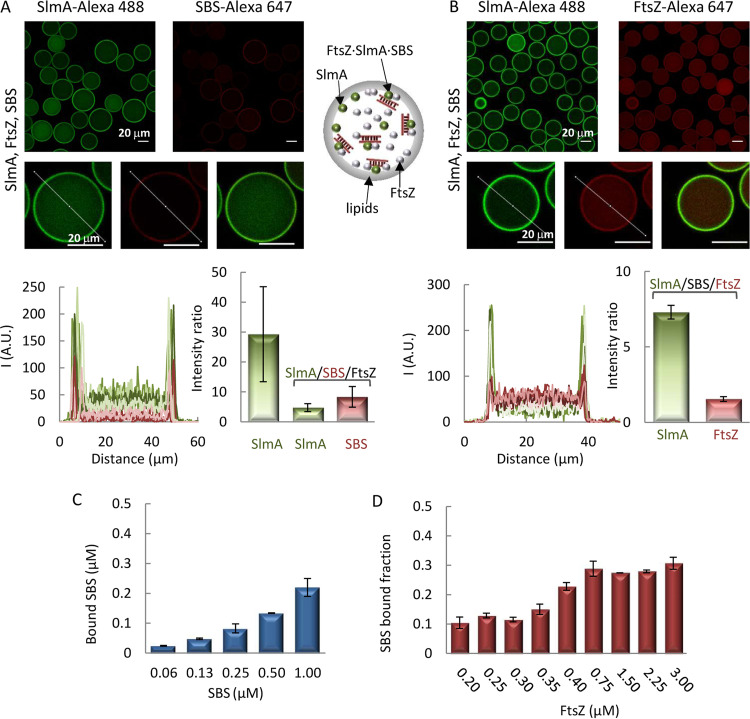
Interaction of SlmA-SBS with lipids in the presence of FtsZ. (A and B) Encapsulation of SlmA, FtsZ, and SBS inside microdroplets stabilized by the E. coli lipid mixture. Shown are representative confocal images and intensity profiles of 5 different microdroplets in the green and red channels obtained across the line depicted in the images, including merged images of single microdroplets. The ratios of the intensity at the membrane to that at the lumen, corresponding to the averages ± SDs, are also shown, together with those of SlmA encapsulated alone under the same buffer conditions, for comparison in panel A. SlmA, FtsZ, and SBS concentrations were 5 μM, 12 μM, and 1 μM, respectively. Tracers concentration was 1 μM. A schematic illustrating the distribution of the three elements in the microdroplets is shown. (C) Variation of the concentration of SBS bound to microbeads coated with E. coli lipids with its total concentration, in the presence of SlmA and FtsZ. SBS was labeled with fluorescein. In all samples, the SBS/SlmA/FtsZ molar ratio was 1:5:12. (D) Variation of the fraction of SBS bound to microbeads coated with E. coli lipids with the concentration of FtsZ. SBS was labeled with fluorescein, and its concentration was 0.125 μM and that of SlmA was 0.5 μM. Negligible binding was obtained at and below 0.1 μM FtsZ. The concentration of lipids for panels C and D was 266 μM. All experiments were in working buffer with 100 mM KCl. Data are the averages from 3 independent experiments ± SDs.

Given the effect of FtsZ on the interaction of SlmA with lipids, we proceeded to characterize its impact on the competition observed between the membrane and the SBS sequences for the protein. For this purpose, the SBS sequence was encapsulated alongside the two proteins in working buffer with 100 mM KCl, and their distribution was assessed by including SlmA-Alexa 488 and one of the other two elements coupled to a red dye as tracers (Fig. 5). In the two types of experiments, SlmA clearly accumulated at the lipid membrane, with a certain amount remaining in the lumen (Fig. 5A and B). The presence of SlmA at nonmembrane regions was higher than when it was the sole species encapsulated (cf. Fig. 5 and 1C) but somewhat lower than when coencapsulated with only one of its partners (Fig. 2 and 4). Images with SBS-Alexa 647 in the presence of FtsZ and SlmA revealed a shift of the DNA toward the lipid boundary, with the consequent appearance of a peak in the red intensity at this location (Fig. 5A). Parallel experiments with FtsZ-Alexa 647 as the secondary fluorescent element revealed its localization both in the lumen and at the lipid boundary, without an obvious preference for any of those locations (Fig. 5B).

We also used E. coli lipid-coated microbead assays to confirm the recruitment of the SBS to the lipids in the presence of SlmA and FtsZ. Incubation of fluorescein-labeled SBS with microbeads in the presence of both SlmA and FtsZ, followed by centrifugation, led to a decrease in the fluorescence signal compatible with sedimentation of the DNA together with the beads (Fig. 5C and D). At constant lipid concentration, the amount of bound DNA increased with its total concentration, in an experiment in which the molar ratio of SBS to SlmA to FtsZ was kept at 1:5:12 (Fig. 5C). Titrations at different concentrations of FtsZ, while maintaining constant those of SBS-Fl, SlmA, and the microbeads, showed an increase in the fraction of SBS recruited to the lipids in parallel with the increase in FtsZ concentration (Fig. 5D). All these experiments indicate that in the presence of FtsZ, SlmA is capable of recruiting the SBS to the membrane.

## DISCUSSION

**FIG 6:**
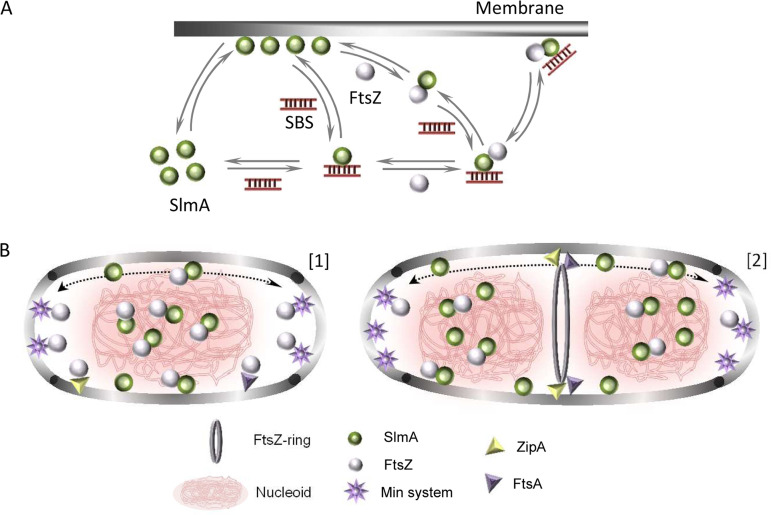
Scheme of the interactions between SlmA, SBS, the membrane, and FtsZ and hypothetical relation with bacterial division stages. (A) SlmA binds to SBS sequences or the membrane in a competitive fashion. The nucleoprotein complexes of SlmA interact with FtsZ, as result of which the two proteins and the DNA gather at the membrane. In the absence of SBS, FtsZ partially dislodges SlmA from the membrane. The partition of SlmA and its complexes at the membrane seems to depend on conditions such as ionic strength and on the relative concentrations of the participating factors (SlmA, FtsZ, SBS sequences, and lipids). (B) Under nondividing conditions (1), SlmA interacts with either the membrane or the SBS sequences within the nucleoid. SlmA and SBS form complexes that may localize at the membrane only upon interaction with FtsZ. Here, additional FtsZ subunits are anchored through their interaction with FtsA and ZipA. The joint action of the Min system and nucleoid occlusion prevents FtsZ ring formation all over the cell. SlmA modulates the oscillation of the Min waves, perhaps when bound to the membrane. (2) Under division conditions, the chromosomes segregate, pulling SlmA and leaving a SlmA free region at midcell where FtsZ, free from the action of the antagonists, now is able to form an FtsZ ring anchored to the membrane by FtsA and ZipA. In noncentral regions, the chromosomes are still protected from aberrant division by SlmA arresting the formation of a ring, and the Min system inhibits its assembly at the poles.

The work presented here shows that the nucleoid occlusion factor SlmA, a protein that specifically targets DNA sequences and engages in complex multivalent interactions with FtsZ, displays membrane binding activity as well. The specific DNA sequence, SBS, has a competing role in this interaction scheme, releasing SlmA from the lipids upon formation of the nucleoprotein complex. FtsZ somehow counteracts this competition and, in its presence, the three elements (FtsZ, SlmA, and the SBS) gather at the lipid membrane (Fig. 6A).

According to our reconstruction experiments, SlmA binds to E. coli lipid minimal membranes (consisting of two kinds of negatively charged lipids and a neutral one) and similarly to model membranes with a single neutral lipid (PC), strongly suggesting that hydrophobic interactions contribute to SlmA lipid binding. This is consistent with the mild impact of salt variations in the 300 mM to 1 M KCl concentration range and with the presence of large hydrophobic regions in SlmA (8). The strong enhancement of membrane binding at 100 mM KCl, however, points toward a direct or indirect influence of electrostatic forces as well. Because this effect is observed for both E. coli lipids and PC, despite their different net charges, it is possible that it arises from modifications in the oligomerization state of the protein itself, its conformation, or the kind of assemblies it might form upon binding to the membrane surface. Sedimentation velocity experiments rule out alterations in the oligomeric state of the protein prior to lipid binding, as the protein profiles retrieved within the 100 to 500 mM KCl interval (see Fig. S6 in the supplemental material) virtually overlap those corresponding to the dimeric species previously described at 300 mM KCl (5). The formation of higher-order SlmA assemblies once on the membrane, promoted by low salt, remains as a possible explanation for the enhanced binding avidity, which would emerge from several transient, probably weak, contacts between SlmA molecules at high local density and the lipid surface. This kind of multivalent interaction appears recurrently in the SlmA interaction network (7). Thus, SlmA targets two low-affinity sites within an FtsZ monomer (10), and stabilization of the overall complexes is achieved through contacts with multiple FtsZ subunits (18) arranged in filaments in the presence of GTP or in shorter oligomers in its absence. Multivalent interactions in the FtsZ-SlmA-SBS complexes are also one of the factors behind their recently observed tendency to reversibly assemble into dynamic biomolecular condensates under crowding conditions (14).

10.1128/mBio.02094-20.6 FIG S6 Effect of KCl on SlmA and SlmA-SBS complexes as determined by sedimentation velocity. Typical sedimentation coefficient distributions of SlmA (5 μM) without and with SBS (1 μM) in working buffer with the specified KCl concentrations. The ∼3 S and ∼6 S single species are compatible with the SlmA dimer and the 4:1 complex formed by SlmA with the DNA, respectively, previously reported (E. J. Cabré, B. Monterroso, C. Alfonso, A. Sanchez-Gorostiaga, et al, PLoS One 10:e0126434, 2015, https://doi.org/10.1371/journal.pone.0126434). Download FIG S6, TIF file, 0.4 MB. Copyright © 2020 Robles-Ramos et al. 2020 Robles-Ramos et al. This content is distributed under the terms of the Creative Commons Attribution 4.0 International license.

The interaction of SlmA with the membrane is modulated by its other natural ligands, FtsZ and the SBS sites, in different ways depending on whether only one of them or both are present (Fig. 6A). Individually, FtsZ and the SBS exhibit a competitive behavior, partially dislodging SlmA from the lipid surface. This behavior radically changes when both of them are present and, under these circumstances, the FtsZ-SlmA-SBS complexes tend to accumulate at the membrane. Competition between SBS sequences and lipids for SlmA could reflect the existence of a common binding site but also a change in the relative orientation, conformation, or stoichiometry induced by each ligand that would preclude recognition by the other. Indeed, SBS interaction with SlmA is known to alter the stoichiometry of the protein, which is otherwise dimeric, in a salt-independent manner (Fig. S6). This is thought to occur with two SlmA dimers in very close proximity and in a highly conserved binding orientation, although not through direct interaction with each other (5, 9). Moreover, SBS-induced conformational changes in SlmA have been also described, such as those stabilizing the flexible DNA binding domain and the binding pocket into which the C-terminal tail of FtsZ gets inserted (19).

It is not obvious why FtsZ is necessary for the simultaneous localization of SlmA and SBS sites at the membrane, but different hypotheses can be formulated. For example, FtsZ may unmask the membrane binding region within SlmA if partially/totally occluded in the SlmA-SBS complexes or induce structural changes more compatible with simultaneous binding of both DNA and membranes. Alternatively, or additionally, the higher multivalency obtained through the interaction with FtsZ may endow the overall complex with properties favoring membrane localization. Further experiments using longer DNAs with a single or multiple SBS sites at different distances, mutants of the two proteins, and GTP to induce FtsZ polymers may help to understand the competitive or synergic effects described here.

Although FtsZ has its specific anchoring proteins, namely, FtsA and ZipA in E. coli (1, 3), the interaction with SlmA and the SBS may also aid in membrane localization of FtsZ, an essential early step in cell division (Fig. 6B). Assembly of these FtsZ units would be under the control of the antagonist, in line with the general thought that to be effective, the negative regulation of FtsZ assembly by SlmA should be exerted not only in the cytoplasm but also next to the membrane (6, 7). Inhibition of FtsZ polymerization is obviously crucial to counteract FtsZ ring formation prior to the start of cell division, but it is still required at other times to prevent aberrant ring formation by the majority of cellular FtsZ that is present outside the central ring (20). Furthermore, as nonring FtsZ seems to form oligomers transiently attached to the membrane (20, 21), inhibition at this location becomes crucial and the cell has developed a robust mechanism based on the action of the Min system. Our results support the idea that SlmA likely acts coordinately with this system at the membrane. Indeed, it was recently proposed that SlmA can modulate the frequency of oscillation of the Min waves, perhaps participating in a conformational change of MinE that determines its interaction with the bacterial membrane (22). Considering our results, it is reasonable to think that besides this putative conformational effect, direct binding of SlmA to the membrane may modify, and in turn may be modified by, the interaction of MinE and the other division components. As neither SlmA nor the Min system uses a sequestration mechanism to inactivate FtsZ polymerization, but instead only shorten the polymers (5, 9, 23), these FtsZ subunits will be available for productive assembly at midcell when required for division (Fig. 6B). The anchoring proteins ZipA and FtsA will aid in releasing FtsZ from these antagonists because they compete for a common target within FtsZ, its C-terminal tail (3).

Membrane recognition has been also reported for the B. subtilis nucleoid occlusion protein Noc, and the concomitant recruitment of DNA seems to be at the heart of the mechanism by which this antagonist blocks aberrant FtsZ ring assembly at noncentral sites (13). Specific interaction with the DNA generates protein clusters that enhance the otherwise low membrane binding tendency of this protein, and exclusion of other divisome proteins by Noc-DNA-membrane complexes locally inhibits division ring assembly. The occurrence of Noc binding sites all over the chromosome except for the Ter macrodomain spatially regulates the formation of the complexes, favoring FtsZ ring assembly at midcell. SlmA may follow a similar mechanism, although in this case, the protein and its specific DNA do not gather at the membrane unless FtsZ is present. Participation of additional proteins in the simultaneous binding of DNA and membranes by Noc cannot be fully excluded in the *in vivo* study reported, but without evidence of interaction with the antagonist, FtsZ does not seem the most obvious candidate (24).

Dual recognition of DNA and membranes also seems to be important for the function of other bacterial proteins, some of which participate in the regulation of cell division. For example, the peripheral membrane protein MinD, which binds to membranes in an ATP-dependent manner, has been also found to bind DNA, suggesting that it could be involved in the overall mechanism of chromosome segregation in addition to its well-known role in the control of division ring positioning (25). Recently, the DNA binding protein MatP, a positive regulator of FtsZ ring assembly that acts indirectly on FtsZ through several FtsZ-binding proteins, has been also found to bind lipid membranes, which may modulate the interplay between chromosome segregation regulation and division site selection by this protein (16). Other bacterial proteins interacting both with membrane surfaces and with DNA sequences include SeqA protein, which sequesters replication origins, the proline utilization A flavoprotein (PutA), and RecA, the SOS repair system regulator (26–28).

Despite recent progress, some of the mechanisms underlying the function of SlmA in cell division still remain enigmatic, reflecting the plasticity of this multivalent factor. The interaction of SlmA with lipid layers introduces another type of binding partner in the pattern of interactions of this protein, and importantly, it provides an additional surface potentially available for the spatial regulation of its function, the inner membrane. These new features and the refined interplay with the other partners and local organizers of the function of this protein, principally the nucleoid and FtsZ, should allow advances in the understanding of the function of this key regulator.

## MATERIALS AND METHODS

### Chemicals and reagents.

Polar extract of E. coli lipids and l-α-phosphatidylcholine from Avanti Polar Lipids (Alabaster, AL, USA) and Sigma, respectively, were dissolved in spectroscopy-grade chloroform and stored at −20°C. Silica microbeads (∼5 μm mean diameter) were purchased from Bangs Laboratories, Inc. (Fishers, IN). Ficoll 70 from GE Healthcare was equilibrated by dialysis in 50 mM Tris-HCl (pH 7.5), 100 mM KCl, and the final concentration was calculated from its refractive index increment (29). Alexa Fluor 488 and Alexa Fluor 647 carboxylic acid succinimidyl ester dyes were from Molecular Probes/Thermo Fisher Scientific. Analytical-grade chemicals were from Merck.

### Protein purification, labeling, and DNA hybridization.

SlmA and FtsZ were purified and subsequently labeled at their amino groups with Alexa Fluor 488 and Alexa Fluor 647, respectively, as previously described (4, 5, 30, 31) and stored at −80°C until used. Ratios of labeling between 0.3 and 0.6 mol of fluorophore per mole of protein were obtained, as calculated from the molar absorption coefficients of the proteins in the UV and the coefficients for the fluorophores in the visible provided by the manufacturer. For the experiments, protein solutions were equilibrated at the specified KCl concentration in 50 mM Tris-HCl (pH 7.5), 5 mM MgCl_2_ (working buffer). Double-stranded DNA with the SBS sequence 5′-AAGTAAGTGAGCGCTCACTTACGT-3′ (bases recognized by SlmA are underlined [4]) was obtained after hybridizing high-pressure liquid chromatography (HPLC)-purified complementary oligonucleotides from IDT or Invitrogen, either labeled with fluorescein or Alexa Fluor 647 at the 5′ end or unlabeled, as described (5).

### Generation of microdroplets and giant vesicles.

Microfluidics chips fabrication and lipid preparation were performed as described previously (16, 32). Oil phase consisted of 20 g/liter E. coli lipids in mineral oil, while aqueous phases varied in composition depending on the experiment (either 5 μM SlmA in both, or 10 μM SlmA in one stream and 2 μM SBS with or without 24 μM FtsZ in the other). The final concentration of SBS (1 μM) was selected to provide a sufficiently strong signal, when labeled, in the confocal images, and the concentration of SlmA was 5 times higher to facilitate formation of the 4:1 SlmA-SBS complexes (5, 9). We chose to use an FtsZ concentration of 12 μM, similar to that used in other *in vitro* studies of FtsZ but ∼2-fold higher than FtsZ levels *in vivo*, in order to mimic the natural excess of FtsZ over SlmA in the cell. Results from the microfluidics experiments agree with parallel experiments using other methods at different concentrations of the two proteins and the SBS. SlmA-Alexa 488, SBS-Alexa 647, and FtsZ-Alexa-647 at 1 μM final concentrations of the labeled species, calculated from the estimated labeling ratios, were used as tracers to visualize each component. Encapsulation was achieved by mixing the two aqueous streams at a 1:1 volume ratio prior to droplet formation, reaching final flows of 135 μl/h (oil phase) and 20 μl/h (total aqueous phase). For the production of giant unilamellar vesicles (GUVs), microdroplets containing 5 μM SlmA with 150 g/liter Ficoll were collected for 30 min in oil phase and centrifuged to force its passage toward an aqueous phase through the coated interface with oriented lipids (33).

### Visualization by confocal microscopy and image analysis.

Microdroplets and vesicles were visualized using a Leica TCS SP5 inverted confocal microscope with an HCX PL APO 63× oil immersion objective (numerical aperture [NA],1.4; Leica, Mannheim, Germany) equipped with 488-nm and 633-nm laser lines essentially as described in reference 33. Intensity profiles of droplets and GUVs were retrieved using the straight-line tool of ImageJ (National Institutes of Health, USA). For each condition, 5 intensity profiles are plotted, corresponding to different microdroplets. The intensity ratios were calculated from the profiles by dividing the average of the intensities at the two edges of a microdroplet by the average intensity in the lumen. Data shown correspond to the average intensity ratio ± standard deviation (SD) from 5 different microdroplets, except for SlmA alone in buffer with 100 mM KCl, for which 15 different droplets were analyzed.

### Binding assays with lipid-coated microbeads.

Microbead lipid-coating procedure, binding measurements, and theoretical estimation of the accessible lipid concentration were performed as stated previously (17). Microbeads were coated with either l-α-phosphatidylcholine or E. coli lipids. The binding of 0.25 μM SlmA-Alexa 488 (with 0.5 mol of dye/mole of protein) to a variable concentration of coated silica microbeads (with either lipid composition) was measured at different KCl concentrations. In the experiments with fluorescein-labeled SBS, FtsZ, and SlmA, the concentration of microbeads was constant (150 g/liter, 266 μM accessible lipids) while varying the concentration of the three elements (keeping the FtsZ/SlmA/SBS-Fl ratio at 12:5:1) or varying only FtsZ (at constant 0.5 μM SlmA and 0.125 μM SBS-Fl). Reported values are the averages from 3 (E. coli lipids) or 2 (PC) independent experiments ± SDs.

Binding isotherms were analyzed or simulated, using user-written scripts and functions in MATLAB (ver. 7.10; MathWorks, Natick, MA), by nonlinear least-squares fit of a Langmuir adsorption isotherm to the experimental data:

y=ymax(cc501+(cc50))

where *y* is the fraction of bound protein and *y*_max_ its maximum value (constrained to 1), *c* is the concentration of accessible lipids, and *c*_50_ is the concentration of them at which half of the maximum fraction of bound SlmA is obtained.

### Fluorescence anisotropy-based competition experiments.

Competition between lipids and the SBS for SlmA was analyzed by fluorescence anisotropy measurements coupled to microbead binding assays using a protocol similar to that described elsewhere (16). Briefly, E. coli lipid-coated microbeads at variable concentrations were incubated with the nucleoprotein complexes in working buffer with 100 mM KCl (0.125 μM SlmA and 63 nM SBS-Fl) or 300 mM KCl (0.25 μM SlmA and 63 nM SBS-Fl), and the anisotropy of the supernatant was measured after low-speed centrifugation of the samples to pellet the microbeads and any element adsorbed. The increase in anisotropy with respect to that measured for the free SBS-Fl, due to SlmA binding, was represented. Reported values correspond to the averages from three independent experiments ± SDs. Equivalent results, within error, were obtained when adding the preformed SlmA-SBS complex to the lipid-coated microbeads or SlmA to a mixture of lipids and SBS.

### Biolayer interferometry measurements.

Detection of lipid-protein interaction by biolayer interferometry was performed using a single-channel BLItz system (ForteBio). E. coli lipids were immobilized on aminopropylsilane biosensor tips using freshly prepared small unilamellar vesicles at 0.5 g/liter (34). The change in the interferometry signal after immersion of the lipid-coated tip in the solution with the protein was recorded in duplicates at each SlmA concentration at room temperature.

### Analytical ultracentrifugation experiments.

Sedimentation velocity profiles of 5 μM SlmA with or without the SBS (1 μM) in working buffer at 100, 300, or 500 mM KCl were obtained after centrifugation at 48,000 rpm and 20°C in an Optima XLA analytical ultracentrifuge (Beckman-Coulter Inc.) equipped with UV-visible (UV-Vis) and Raleigh interference system. For the experiments, an An-50Ti rotor and 12-mm double-sector centerpieces were used. Sedimentation coefficient distribution was calculated from the absorbance signal at 230 nm by least-squares boundary modeling of sedimentation velocity data using the c(s) method implemented in SEDFIT (35), and the calculated *s* values were corrected to standard conditions (water, 20°C, and infinite dilution) using SEDNTERP (36).
