# Supplementary figures and images for: The Nucleoid Occlusion Protein SlmA Binds to Lipid Membranes
Source: mBio. 2020 Sep 1;11(5):e02094-20. doi: 10.1128/mBio.02094-20 (PMC7468209; doi:10.1128/mBio.02094-20)

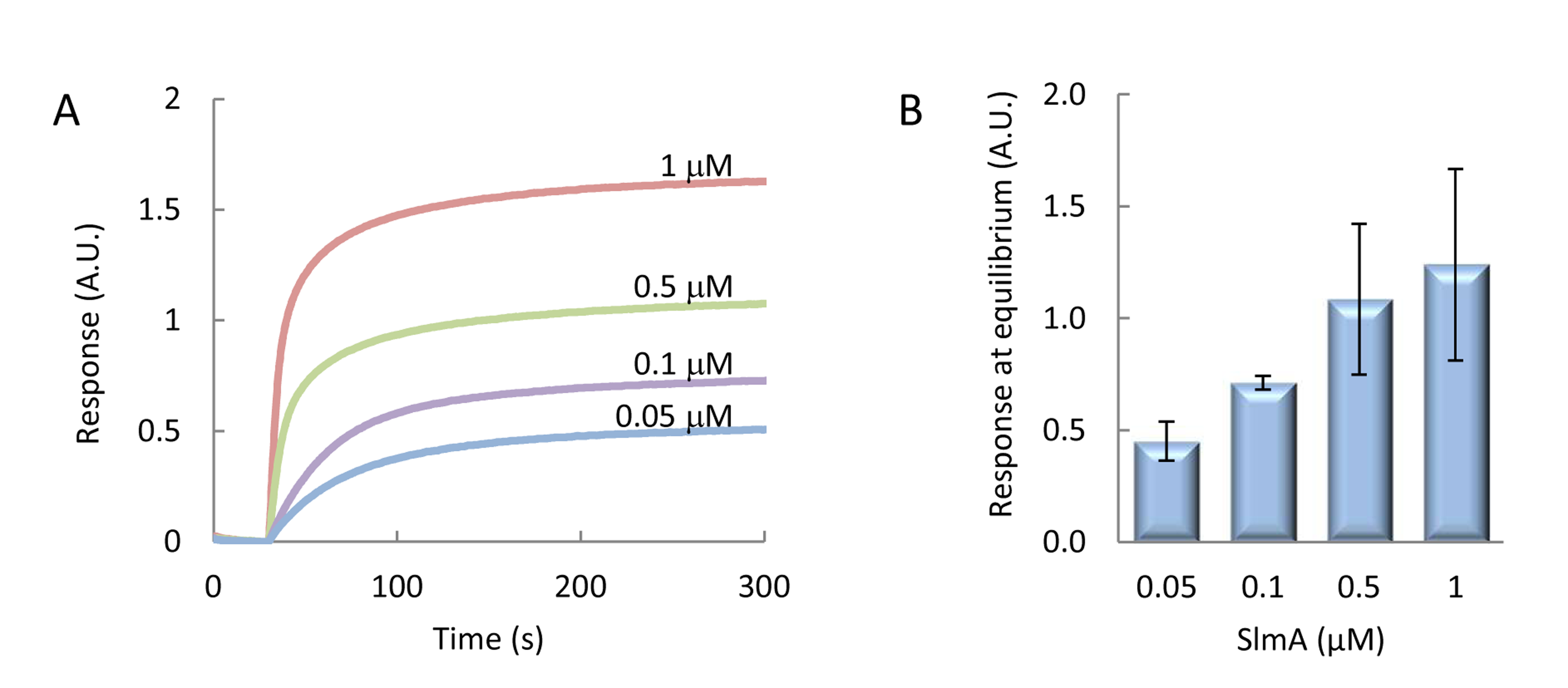

Supplement: FIG S1 [file mBio.02094-20-sf001.tif]

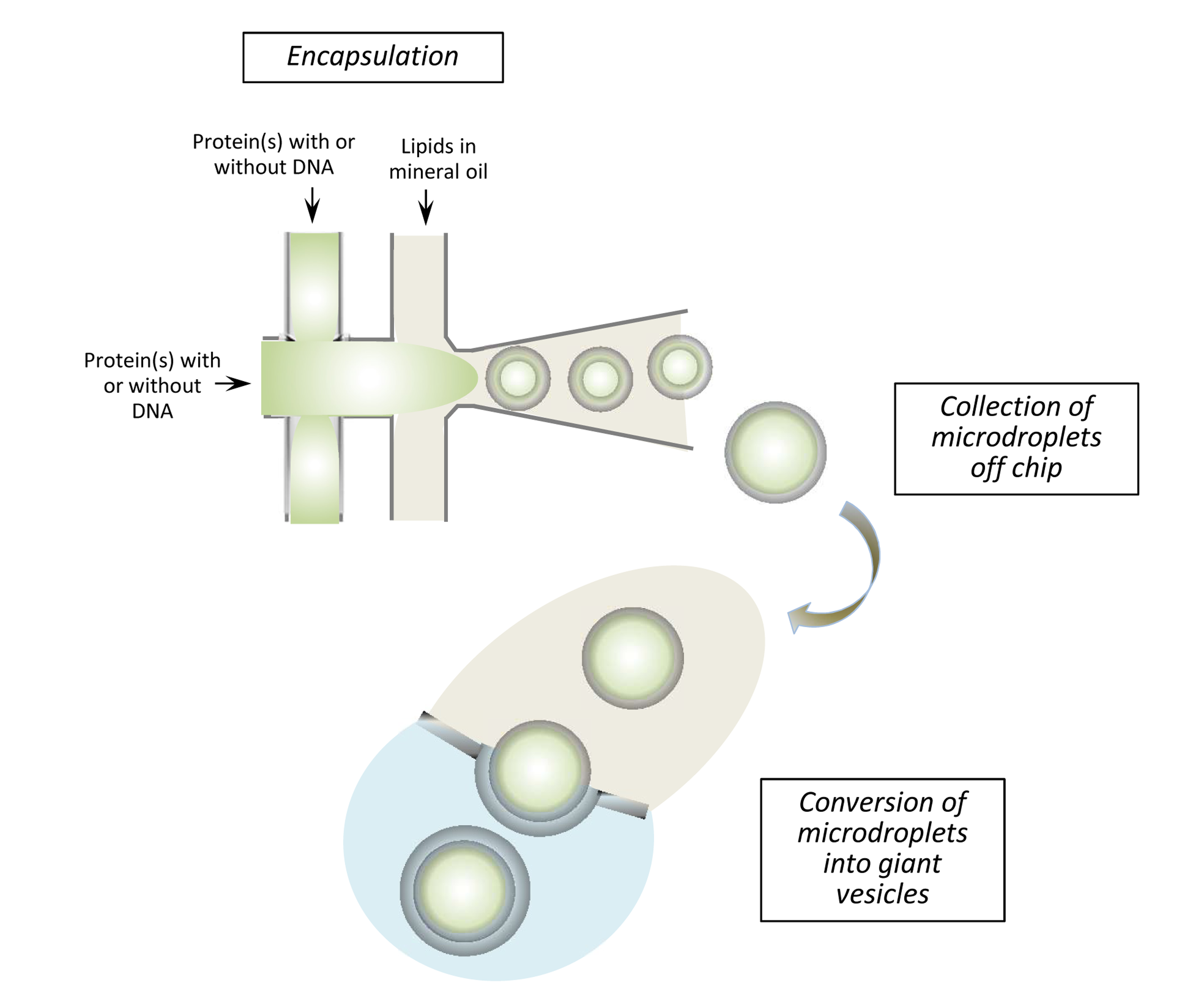

Supplement: FIG S2 [file mBio.02094-20-sf002.tif]

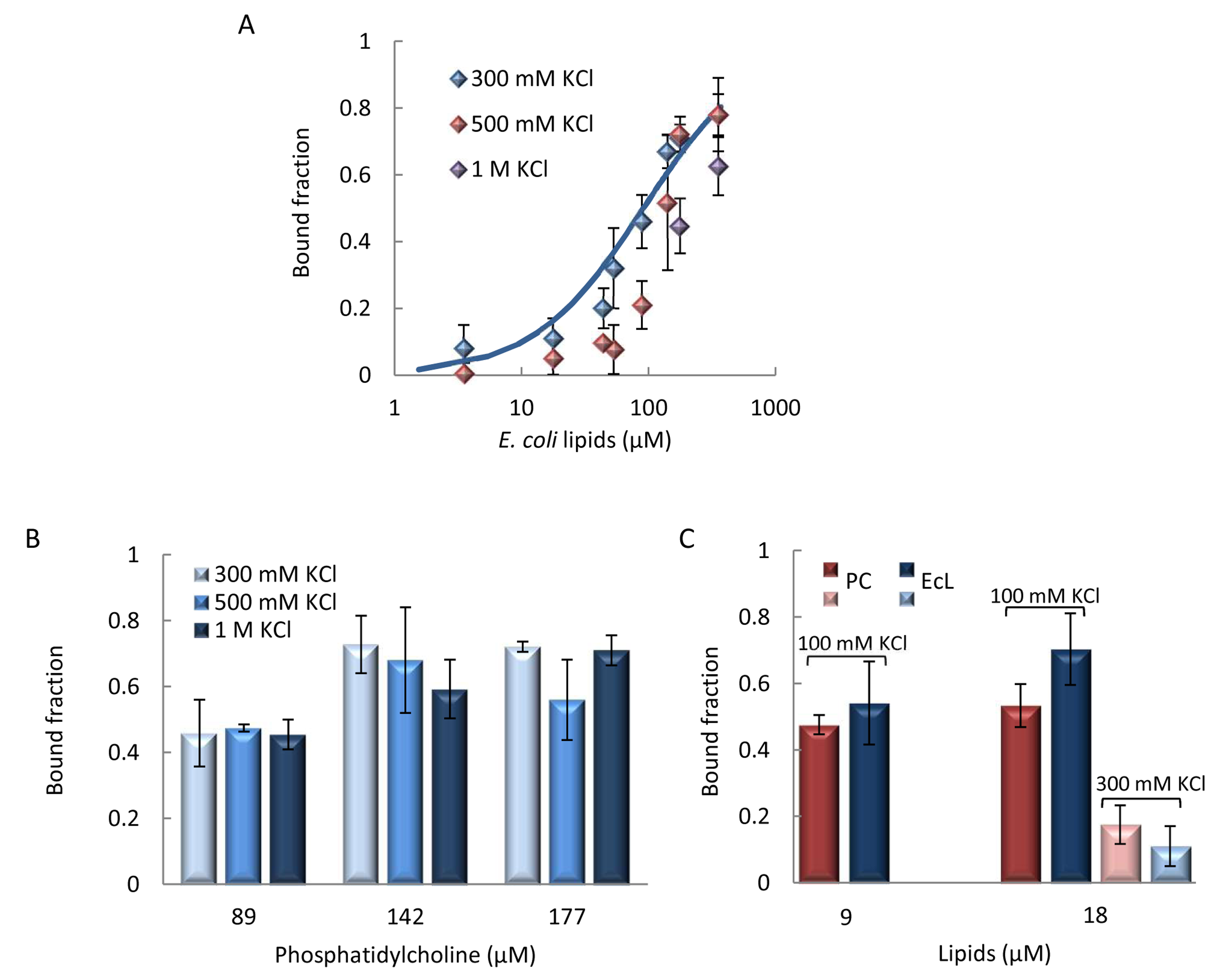

Supplement: FIG S3 [file mBio.02094-20-sf003.tif]

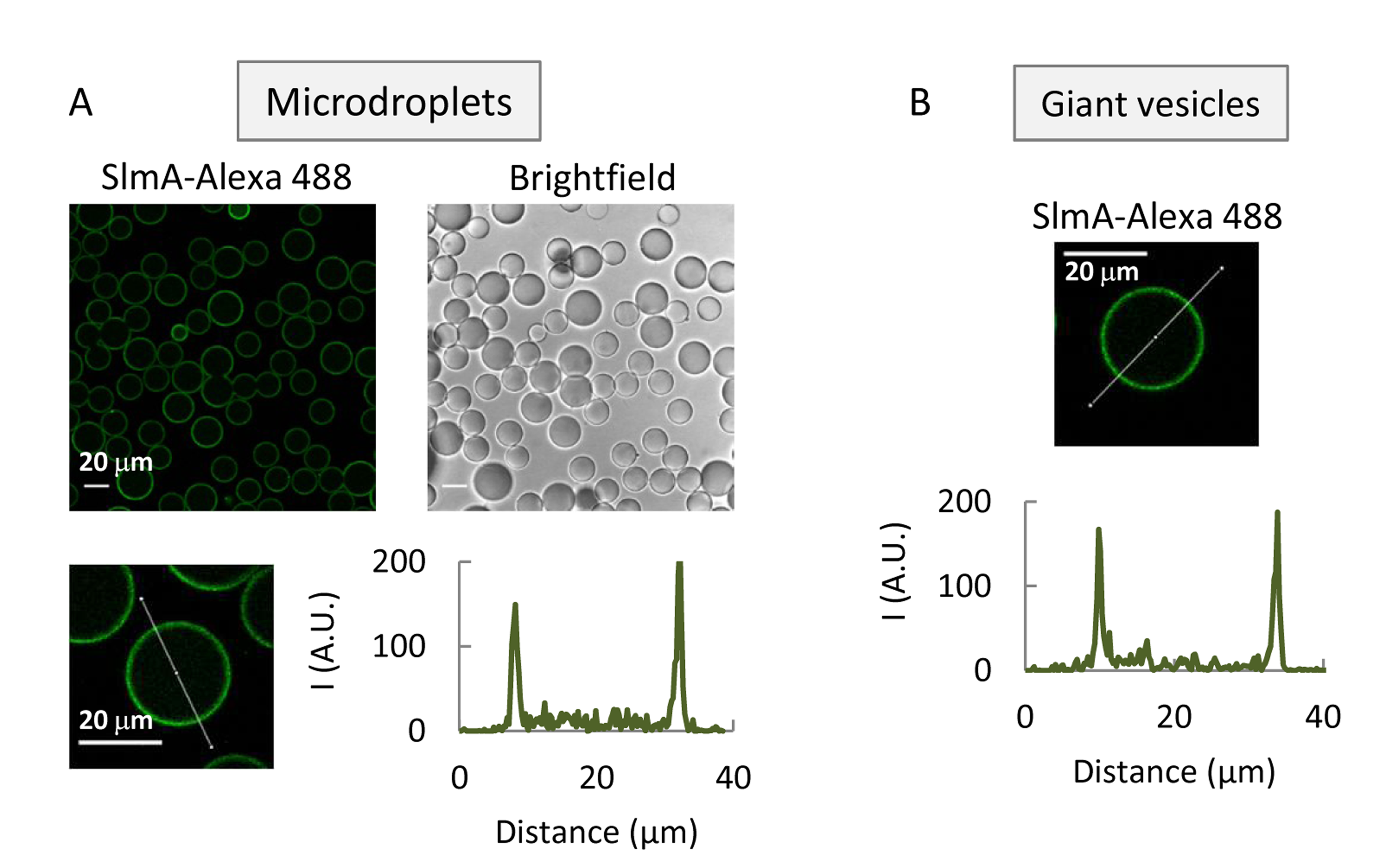

Supplement: FIG S4 [file mBio.02094-20-sf004.tif]

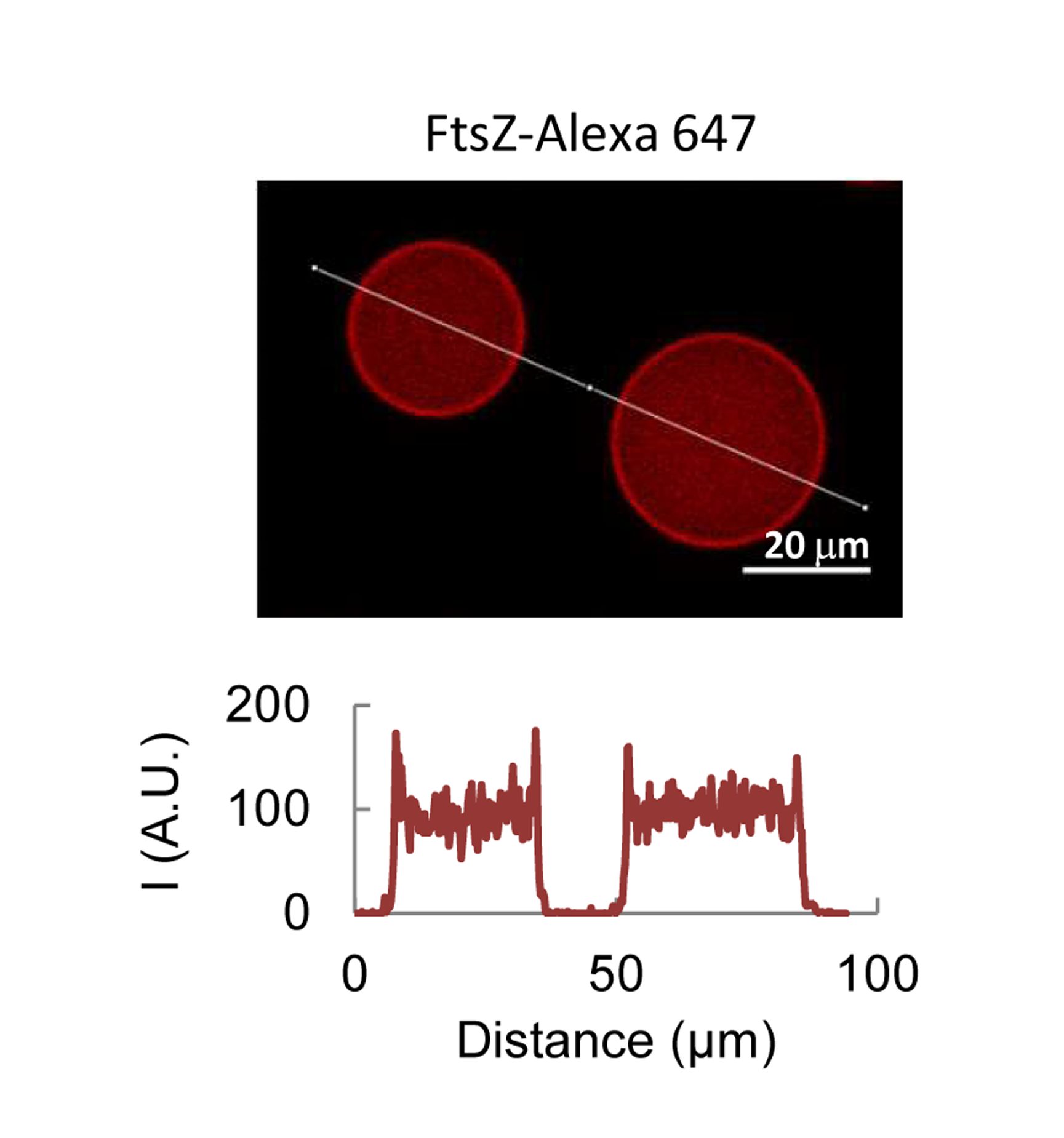

Supplement: FIG S5 [file mBio.02094-20-sf005.tif]

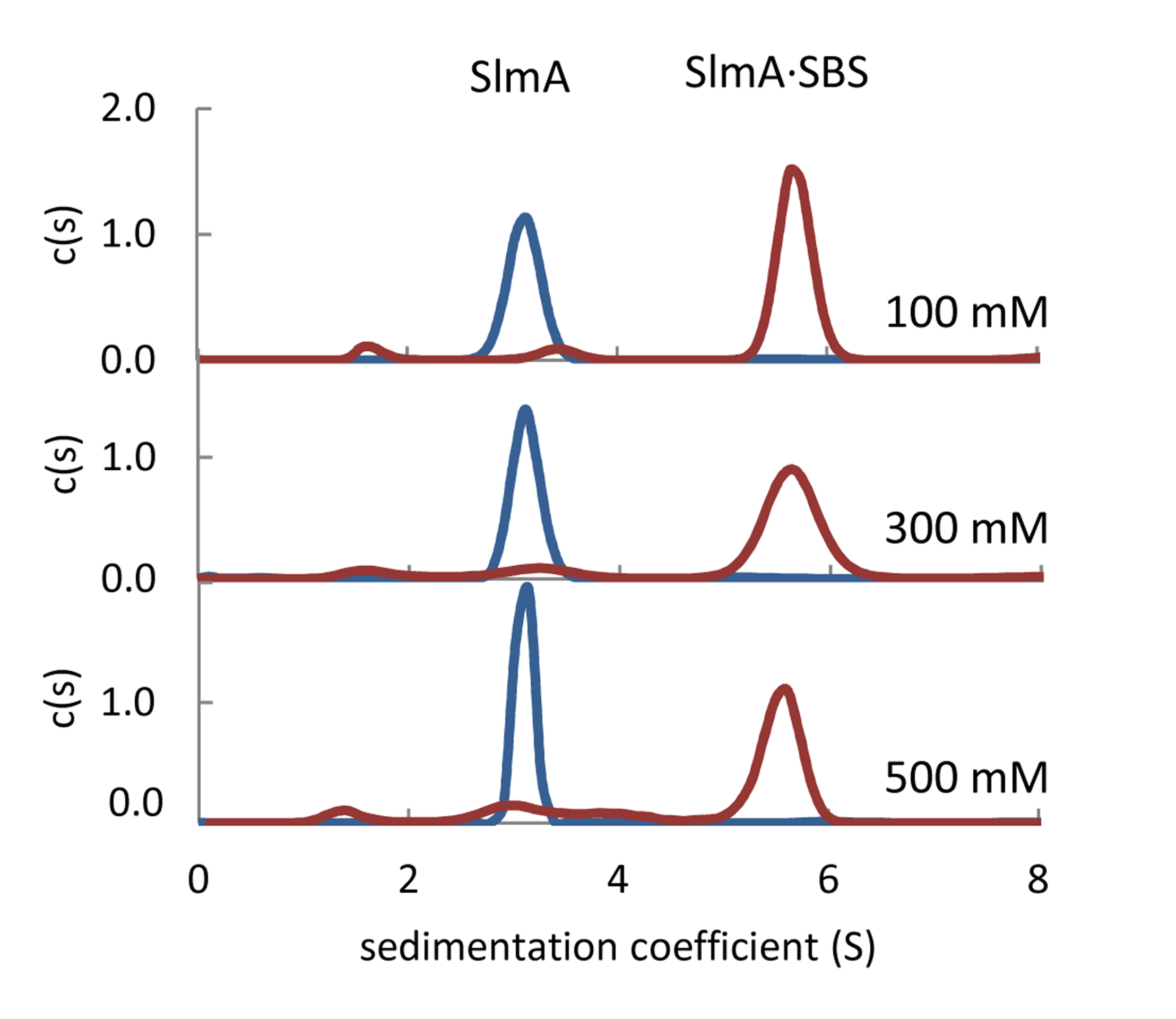

Supplement: FIG S6 [file mBio.02094-20-sf006.tif]
